# Stress-induced pro- and anti-inflammatory cytokine concentrations in female PTSD and depressive patients

**DOI:** 10.1038/s41398-022-01921-1

**Published:** 2022-04-14

**Authors:** Vanessa Renner, Julia Schellong, Stefan Bornstein, Katja Petrowski

**Affiliations:** 1 Medical Psychology & Medical Sociology, University Medical Center of the Johannes Gutenberg University Mainz, Mainz, Germany; 2 University Medical Center Carl Gustav Carus, Technische Universität Dresden, Dresden, Germany

**Keywords:** Depression, Predictive markers

## Abstract

Alterations of the hypothalamus pituitary-axis on one hand and heightened rates of somatic diseases and mortality on the other hand are consistently found for PTSD and MDD patients. A possible link between these factors might be the immune system, in particular pro- and anti-inflammatory cytokines. A ‘low-grade inflammation’ in PTSD and MDD patients was found, whereas the influence of acute stress and the role of anti-inflammatory cytokines was rarely examined. In this study, 17 female PTSD patients participated in the Trier social stress test while serum cytokine levels (IL-6, IL-10) were assessed. Cytokine levels of PTSD patients were compared with levels of female depressive patients (*n* = 18) and female healthy controls (*n* = 18). Group differences were assessed using a 3 (group) x 8 (time: −15, −1, +1, +10, +20, +30, +45, +60 min) ANCOVA for repeated measures with baseline values as covariates. There was no group difference regarding IL-6 levels (*p* = 0.920) but PTSD patients showed significantly higher levels of IL-10 compared with depressive patients (*p* < 0.001, *d* = 0.16) and healthy controls (*p* = 0.001, *d* = 0.38). Under acute stress, PTSD patients did not show the widely found elevated IL-6 levels but showed an increase of anti-inflammatory IL-10. Therefore, acute stress seems to promote an imbalance of pro- and anti-inflammatory cytokine levels in PTSD and might indicate a hyperreactive immune response. This should be considered in future studies to further understand the role of the immune system as a link between stress response and somatic diseases.

## Introduction

Patients suffering from mental disorders show higher rates of health care utilization, cardiac events, cardiac mortality and show higher mortality rates in general [1–4**]**. These effects are still present when controlling for other risk factors such as smoking history, socioeconomic status, Body Mass Index or somatic conditions such as hypertension [1, 3]. Posttraumatic stress disorder (PTSD) and major depressive disorder (MDD) are among the most common mental disorders with 6–9% of the European and American population suffering from PTSD and 12–15% suffering from depression [5]. Both are associated with a range of different somatic diseases such as cardiovascular, respiratory, gastrointestinal and autoimmune diseases [1, 4, 6, 7]. In general, female MDD and PTSD patients showed a 4-fold higher mortality risk compared with individuals not suffering from mental disorders even after controlling for other risk factors [4].

These findings emphasize the possibility of a direct association between psychological and somatic diseases. A possible explanation for these findings is an alteration of the hypothalamus pituitary adrenal-axis (HPA-axis) of PTSD and MDD patients which can in turn influence the immune system via cytokines [8]. Cytokines, a family of proteins mediating immune responses to injury, infection or other organismal stress, are associated with inflammatory diseases such as cardiovascular diseases, cancer, pulmonary disease or autoimmune disorders [8, 9]. An activation of the HPA-axis because of stress leads to a secretion of corticotrophin-releasing factor (CRF) from the hypothalamus [10]. CRF stimulates the release of adreno-corticothrophic hormone (ACTH) and this in turn stimulates the release of glucocorticoids. Glucocorticoids are hormones which can inhibit inflammatory cytokines such as Interleukin (IL-) 6 by downregulating cytokine expression and increase production of anti-inflammatory cytokines such as IL-10 via T cells. Thereby, an immunoregulatory activity is possessed [10]. These mechanisms are supposed to be altered in mental disorders and might therefore lead to body states promoting the development of the mentioned somatic diseases. Though different studies investigated the reactivity of the HPA-axis and cytokine levels in different patient groups, the influence of acute stress on cytokine levels in PTSD and MDD patients still remains unclear.

By assessing cortisol levels, conclusions about the HPA-axis response can be drawn. In a standardized stress test, a blunted HPA-axis stress reactivity was found in female PTSD patients compared with healthy controls [11]. Also, in a meta-analysis, lower cortisol levels in plasma and serum of PTSD patients were found compared with healthy controls [12]. Regarding cortisol reactivity of MDD patients, they showed descriptively lower levels than healthy controls and significantly lower levels than PTSD patients [13]. In general, PTSD and MDD patients show altered cortisol responses to stressors [11–15].

These alterations of the HPA-axis could influence the immune response and in turn lead to more physical diseases in these patient groups. In PTSD, higher levels of pro-inflammatory markers compared with healthy controls were found, leading to the assumption of a ‘low-grade inflammation’ in PTSD [8, 9, 16]. Also, in MDD patients elevated levels of pro-inflammatory cytokines such as IL-6 were found compared with healthy controls [17, 18]. Therefore, it is suggested that these inflammatory states in PTSD and MDD might be a psychobiological mediator between psychological and somatic diseases [14]. Nevertheless, the influence of acute stress on cytokine levels in PTSD and MDD and the relation of cytokine levels in these disorders was rarely examined.

In addition to the low number of studies assessing cytokine levels of these patients under acute stress, the role of inflammatory cytokines such as IL-1β or IL-6 are mainly investigated and the role of anti-inflammatory cytokines such as IL-10 tends to be neglected.

To further assess cytokine levels of PTSD and MDD patients under acute stress, we assessed IL-6 and IL-10 levels of these patient groups and of healthy controls during the Trier social stress test. Based on this, conclusions about the acute stress reaction of these patients compared with healthy controls can be drawn. Additionally, conclusions about the cytokine levels of PTSD patients in relation to levels of MDD patients can be drawn. With this approach this study provides further insights regarding the assumed HPA-immune interaction via cytokines.

## Methods

### Study sample

Patient groups (PTSD, MDD) were recruited at the University Medical Center Carl Gustav Carus of the Technische Universität Dresden during the beginning of their inpatient treatment or treatment at the day clinic of the Clinic and Policlinic for Psychotherapy and Psychosomatics. Healthy controls were recruited via announcements at the website of the clinic. Participants with fluent German language skills and age between 18 and 65 were included in the study. General exclusion criteria were a lifetime history of substance use disorder, psychotic or bipolar disorder, psychopharmacological or glucocorticoid-containing medication intake (e.g. asthma inhaler, topical cortisone creams), severe medical illnesses (e.g. cancer, autoimmune diseases, diabetes), and pregnancy. The Structured Clinical Interview (SCID-IV) [19] was conducted by trained interviewers for the assessment of *DSM-IV-TR* mental disorder diagnoses. Patients with a primary diagnosis of PTSD were included and assigned to the PTSD group, patients with a primary diagnosis of MDD were assigned to the MDD group. Healthy control group participants required to be free of any history of mental disorder in the SCID.

The final sample included *n* = 17 PTSD patients, *n* = 18 MDD patients and *n* = 18 healthy controls. The groups were matched regarding gender and age. None of the participants did use contraceptives. In the PTSD group, *n* = 16 additionally suffered from MDD, *n* = 6 from panic disorder with/ without agoraphobia, *n* = 9 from social anxiety disorder, *n* = 8 from somatoform disorder. In the MDD group, *n* = 4 patients additionally suffered from panic disorder with/ without agoraphobia. All the study participants provided written informed consent and the study procedure was conducted in accordance with the Declaration of Helsinki and approved by the local Ethics Committee of the Medical Faculty of the Technische Universität Dresden, Germany.

### Psychosocial stress induction and hormone sampling

The standardized protocol for the TSST was applied for the reliable induction of acute moderate psychosocial stress under laboratory conditions [20, 21]. In brief, the TSST requires a mock job interview (5 min) and a mental arithmetic task (5 min) of the participants to be performed in front of a mock selection committee. Women were tested exclusively in the luteal phase of their menstrual cycle. To account for the circadian rhythm of cytokine secretion, the TSST was performed not earlier than 2 pm in the afternoon. Subjective levels of distress were evaluated using the Primary Appraisal Secondary Appraisal Questionnaire (PASA) [22] prior to the TSST and a Visual Analogue Scale (VAS) following the TSST. Blood samples were collected via a venous catheter 15 min and 1 min prior to the TSST as well as 1, 10, 20, 30, 45 and 60 min after the TSST and set to coagulate for 30 min at room temperature. The blood samples were then centrifuged at 20 °C for 10 min at 2500 x G RCF. Blood samples are stored at −80 °C before being assayed for cytokines. Serum cytokine concentrations (IL-6 and IL-10) were determined using highly-sensitive ELISA enzyme-linked immunosorbent assays (IBL International GmbH, Germany). The detection limit was <0.04 pg/ml.

### Clinical assessment

Three German language self-report questionnaires were handed out to the study participants for a clinical characterization. Overall, higher questionnaire scores indicate higher disease severity. (1) The Symptom Checklist (SCL-90-R) [23, 24] assesses the general psychological symptom burden within the last seven days. The questionnaire consists of 90 items. The global severity index (GSI) reflects the degree of general impairment. The SCL-90-R meets high internal consistencies with Cronbach’s α = 0.97 for the global index. (2) The Impact of Event Scale (IES-R) [25, 26] assess typical symptoms in consequence of traumatic events. The questionnaire contains 22 items which load on three subscales: intrusions, avoidance and hyperarousal. Internal consistencies are quite high with Cronbach’s alpha α = 0.79 to 0.90. (3) Depressiveness was evaluated with the Beck Depression Inventory II (BDI-II) [27, 28] including 21 items that match the *DSM-IV-TR* major depression criteria. The BDI-II shows high internal consistency with Cronbach’s alpha α = 0.93.

Subjective levels of distress were evaluated using the Primary Appraisal Secondary Appraisal Questionnaire (PASA) [22] prior to the TSST. With the PASA, general stress-related cognitions are assessed. On the primary appraisal scale, the threat of the current situation is rated, on the secondary appraisal scale individuals rate possible coping mechanisms for this situation. Based on these scales, the general stress index is calculated. Internal consistencies (Cronbach’s alpha) α = 0.61 to 0.83 [22]. A Visual Analogue Scale (VAS) was presented following the TSST including 8 questions regarding the experiences during the TSST (e.g. “The situation was stressful for me”, “I experienced the situation as a challenge”).

### Statistical analyses

A power analysis was conducted with StataIC 16 V5 to calculate an appropriate sample size. Therefore, based on a power of 80%, α = 0.05, 8 repetitions and three groups (PTSD, MDD, HC) were assumed and matrices including expected effects per time point per group were used for calculations. Based on the literature, HCs were assumed to show smaller effects than both patient groups. Based on this calculation, a sample size *N* = 51, resulting in *n* = 17 per group was proposed and implemented. All further analyses were performed using SPSS, version 27 (IBM, Chicago, Illinois). Group comparisons with respect to sociodemographic and clinical variables were evaluated using univariate analyses of variance (ANOVA) for continuous variables and Chi-square test (*χ*^2^) for dichotomous variables. The distributions of all variables were evaluated prior to analyses. Because IL-6 and IL-10 values were skewed, they were subjected to ln-transformations [29]. Missing cytokine values were imputed using predictive means matching over 10 iterations using R with the package *mice* [30]. Group differences in cytokine levels (IL-6, IL-10) were assessed using a 3 (group: PTSD patients, MDD patients, healthy controls) x 8 (time: −15, −1, +1, +10, +20, +30, +45, +60 min) ANCOVA for repeated measures with baseline values (−15, −1 min) as covariates to control for baseline differences between groups and Greenhouse-Geisser corrections, if necessary. Statistical differences were considered significant with *p* < 0.05.

## Results

The groups were matched by gender (100% female) and age, leading to no significant differences regarding age (*M*-PTSD = 46.88, *SD*-PTSD = 8.40; *M*-MDD = 38.61, *SD*-MDD = 13.65; *M*-HC = 41.94, *SD*-HC = 12.17; *χ*^*2*^ = 4.01, *p* = 0.134). Means and standard deviations of GSI (SCL-90-R), BDI-II, IES-R, PASA and VAS values as well as group differences regarding these values can be derived from Table 1. As expected, groups did differ regarding subjective symptom burden. Most importantly, both patient groups did show higher scores on the GSI compared with healthy controls. PTSD patients reported more symptoms of intrusion, hyperarousal and avoidance compared with MDD patients and healthy controls and PTSD as well as MDD patients reported more depressive symptoms on the BDI-II than healthy controls. There were no significant group differences regarding PASA or VAS.

**Table 1:** Means, standard deviations of questionnaire data and differences between groups (ANOVA with Post hoc-tests, Bonferroni corrected).

	PTSD *Mean (SD)*	MDD *Mean (SD)*	HC *Mean (SD)*	*M-Diff* PTSD-MDD (*d*)	*M-Diff* PTSD-HC (*d*)	*M-Diff* MDD-HC (*d*)
GSI	41.75 (4.06)	36.29 (4.27)	28.72 (5.80)	0.10** (1.31)	0.16** (2.59)	0.06* (1.49)
IES-R: intrusion	21.13 (11.36)	6.07 (5.66)	3.78 (4.49)	14.93** (1.69)	17.22** (2.03)	2.29
IES-R: avoidance	17.50 (4.04)	5.07 (6.01)	3.44 (4.78)	11.82** (2.41)	13.44** (3.17)	1.63
IES-R: hyperarousal	17.63 (9.13)	3.42 (4.51)	2.56 (3.88)	14.57** (1.99)	15.44** (2.17)	0.87
BDI-II	28.88 (7.97)	13.57 (9.25)	6.28 (6.12)	14.70** (1.77)	23.87** (3.19)	9.17* (0.93)
VAS	53.70 (7.36)	50.36 (13.99)	54.77 (11.85)	3.35	−1.07	−4.42
PASA	–0.50	–0.06	–1.61	–0.44	1.11	1.56
Stress index	(4.73)	(3.84)	(4.15)			

*PTSD* post traumatic stress disorder, *MDD* major depression disorder, *HC* healthy control, *SD* Standard deviations, *M-Diff* Mean difference, *GSI* Global Severity Index, *IES-R* impact of event scale, *BDI-II* Beck depression inventory, *VAS* visual analog scale, *PASA* primary appraisal secondary appraisal, d effect size Cohen’s d.

*p < 0.01; **p < 0.001.

**Fig. 1:**
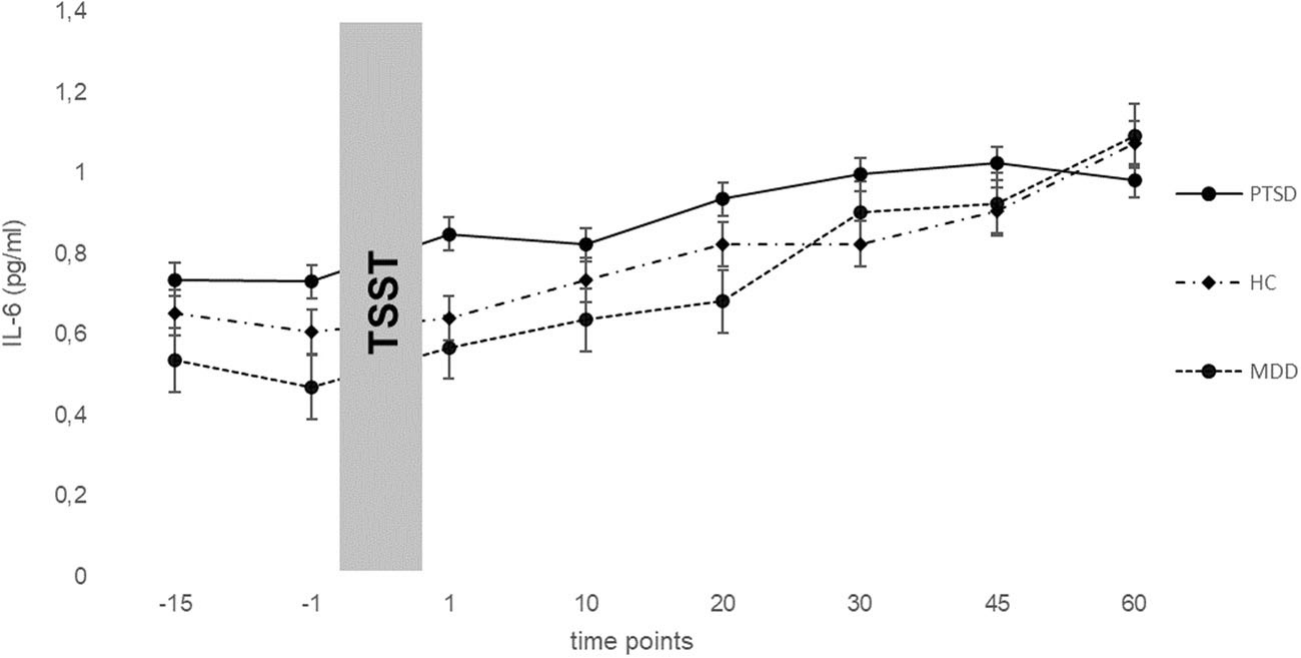
Mean (±SE) IL-6 levels across measurement points of the TSST in PTSD, MDD patients and HCs. IL-6 Interleukin-6, TSST trier social stress test, PTSD post traumatic stress disorder, HC healthy controls, MDD major depression disorder, time points in minutes.

**Fig. 2:**
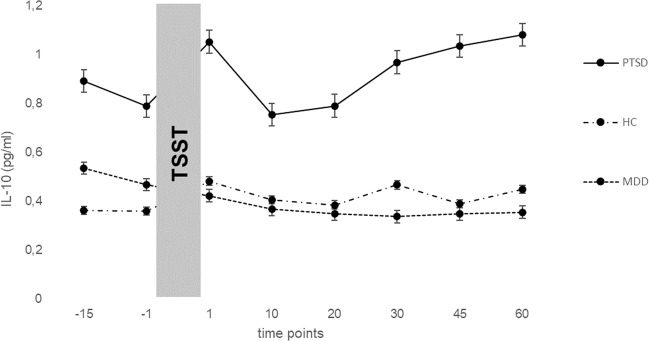
Mean (±SE) IL-10 levels across measurement points of the TSST in PTSD, MDD patients and HCs. IL-6 Interleukin-6, TSST trier social stress test, PTSD post traumatic stress disorder, HC healthy controls, MDD major depression disorder, time points in minutes.

Figures 1 and 2 show mean values of IL-6 and IL-10 levels for the measurement points of the TSST of PTSD, MDD patients and healthy controls. Results of the repeated measures ANCOVA can be derived from Table 2. Comparing IL-6 values, there was a significant main effect for time (*F* (3.94, 189.25) = 9.03, *p* < 0.001, *η*_p_^2^ = 0.158) but not for group (*F* (2, 48) = 0.08, *p* = 0.920). IL-10 levels showed significant main effects for time (*F* (3.80, 182.20) = 4.88, *p* = 0.001, *η*_p_^2^ = 0.092), a significant main effect for group (*F* (2, 48) = 15.22, *p* < 0.001, *η*_p_^2^ = 0.388) and a significant interaction for time x group (*F* (7.59, 182.20) = 3.05, *p* = 0.004, *η*_p_^2^ = 0.113). Bonferroni-adjusted post hoc analysis revealed significant differences in IL-10 levels between PTSD and MDD patients (0.38, *p* < 0.001, 95%-CI [0.21; 0.55]; *d* = 0.16) as well as between PTSD patients and healthy controls (0.27, *p* = 0.001, 95%-CI [0.09; 0.45]; *d* = 0.38). There was no significant difference between MDD patients and healthy controls (0.11, *p* = 0.214, 95%-CI [−0.04; 0.26]).

**Table 2:** Results of repeated measures ANCOVA with cytokine baseline-values (−15 min, −1 min) as covariates.

	Sum of Squares	df	Mean Squares	*F*	*p*	*η*^2^_part_
IL-6						
Time	1.99	3.94	0.50	9.03	<0.001	0.16
Group	0.08	2	0.04	0.08	0.92	
Time x Group	0.73	7.89	0.09	1.66	0.11	
IL-10						
Time	0.99	3.80	0.26	4.88	0.001	0.09
Group	5.71	2	2.86	15.22	<0.001	0.39
Time x Group	1.23	7.59	0.16	3.05	0.004	0.11

Values were Greenhouse-Geisser corrected if necessary.

## Discussion

In this study levels of pro- and anti-inflammatory cytokines were assessed under acute stress in PTSD and MDD patients and were compared with healthy controls. There were no group differences regarding pro-inflammatory cytokines (IL-6) but PTSD patients showed higher levels of anti-inflammatory cytokines (IL-10) compared with MDD and healthy controls. There was no difference in IL-10 levels between MDD patients and healthy controls. The finding of no group differences of pro-inflammatory cytokines is in contrast to past studies showing elevated IL-6 levels and suggesting a ‘low-grade inflammation’ in PTSD and MDD [8, 9, 16–18, 31]. In contrast to the present study, these studies mainly assessed fluctuating or spontaneous IL-6 levels without the influence of an acute stressor, which could be one reason for the discrepancies. Acute stress could influence the immune response of PTSD patients in a way that anti-inflammatory IL-10 becomes more present than in healthy controls or MDD patients and not – as found when assessed spontaneously – pro-inflammatory IL-6. Nevertheless, past studies found elevated IL-6 levels in PTSD and MDD [14, 32] patients under acute stress, after the TSST. Other studies reported no difference between PTSD patients and healthy controls regarding IL-6 but did find differences regarding other pro-inflammatory cytokines such as IL-1β and Tumor necrosis factor (TNF) α [33]. They assumed, that these elevated levels might be first steps of a pro-inflammatory cascade later resulting in elevated IL-6 levels. As we did not assess these parameters, we cannot support this assumption with our data.

Elevated levels of IL-10 in PTSD patients compared with healthy controls were also found in past studies when serum cytokine levels were assessed [34, 35]. Higher levels of IL-10 usually occur to reduce inflammatory processes by inhibiting the production of pro-inflammatory cytokines such as IL-6 and therefore protecting the individual from an increased pro-inflammatory state with negative somatic consequences [35, 36]. On the other hand, as described, IL-10 is released via T-cells distinguished in two subtypes: T helper lymphocytes 1 (Th1) and T helper lymphocytes 2 (Th2) both releasing different sets of cytokines [10, 37]. It is suggested that an imbalance of Th1 and Th2 can be triggered by stress and may in turn encourage and/ or sustain different somatic diseases [37, 38]. For example, stress-induced Th1 suppression leads to a Th2-shift, an overproduction of IL-10 amongst others and may favor the development of different cancer types [37, 38]. Based on this, especially a hyperreactive immune response due to an imbalance of Th1/ Th2 could play a crucial role in the development of somatic diseases. As shown, PTSD patients have higher rates of circulating T-cells compared with healthy controls, which might support this assumption and which could lead to a hyperreactive immune response [1]. One assumption could be, that PTSD patients in general may show a hyperreactive immune response with an imbalance of pro- and anti-inflammatory cytokines and that this imbalance depends on the current state in which the individual is (acute stress vs. spontaneous assessment). This assumption needs to be further investigated whereas especially acute stressors could play a major role in the immune response and a potentially associated hyperreactivity promoting somatic diseases. In contrast to the other two groups, IL-10 levels of the PTSD patients show an increase directly after the TSST, decreases quickly and then rises again continuously. As studies assessing IL-10 levels under acute stress are very rare, it is not clear if this is a typical pattern in PTSD patients. Subjective stress levels (PASA, VAS) do not provide an explanation for this IL-10 pattern.

In contrast, MDD patients did not show elevated IL-10 levels compared with healthy controls, which is in line with past studies [31]. As MDD patients do not show any differences in cytokine levels compared with healthy controls, the specific role of the immune system under acute stress in MDD patients remains unclear. The results more hint to the conclusion that other mechanisms might play a more important role in linking MDD and alterations in the HPA-axis with somatic diseases. Nevertheless, as described above past studies did find elevated IL-6 levels in MDD [17, 18], emphasizing alterations of the immune system in this patient group. For further conclusions future studies need to assess pro- and anti-inflammatory cytokine levels of MDD patients under acute stress.

One limitation of our study is, that potentially influencing factors such as severity or type of trauma were not taken into account. Additionally, PTSD patients showed high comorbidity rates. 16 out of 17 PTSD patients also suffered from MDD and PTSD patients showed higher BDI-II scores than MDD patients. Nevertheless, PTSD patients usually also suffer from other psychological diseases which reduces the possibility to assess patients only suffering from PTSD and would affect the generalizability of the results. Additionally, results of questionnaires about subjective symptom burden regarding PTSD symptoms showed, that the group of PTSD patients showed more/ severe symptoms of PTSD compared with MDD which justifies the differentiation of these groups. PTSD patients also showed higher general symptom burden (SCL-90-R) compared with MDD patients showing that PTSD patients in this study generally suffer from more/ severe symptoms. Lindqvist et al. (2014) [39] also showed, that despite influencing factors such as comorbid depression or time since trauma, elevated pro-inflammatory cytokine levels could still be found though it was accounted for these factors. This study investigated two representatives of pro- (IL-6) and anti-inflammatory (IL-10) cytokines. To draw general conclusions about patterns of these cytokines in PTSD and MDD patients under acute stress, in future studies further cytokines need to be taken into account. As only female participants were investigated in this study, the generalizability of the results is reduced. As shown in past studies [40], there are gender differences in the immune response when individuals are under acute stress which emphasizes the necessity of investigating cytokine levels of both female and male PTSD and MDD patients under acute stress.

All in all, this study shows elevated anti-inflammatory cytokine levels in PTSD patients compared with MDD and healthy controls and no elevated levels of pro-inflammatory IL-6 in both patient groups compared with healthy controls. This finding might reflect a hyperreactive immune system in PTSD patients, which could lead to higher rates of somatic diseases and mortality in these patients. This assumption needs to be investigated in future studies wherein the influence of acute stress on cytokine levels should be taken into account.

## Supplementary information

Supplemental Material
