# Supplementary material for: Stress-induced pro- and anti-inflammatory cytokine concentrations in female PTSD and depressive patients
Source: Transl Psychiatry. 2022 Apr 14;12:158. doi: 10.1038/s41398-022-01921-1 (PMC9010431; doi:10.1038/s41398-022-01921-1)
Supplement: Supplementary file 1 — Supplemental Material [file 41398_2022_1921_MOESM1_ESM.docx]

**Supplementary material**

Table 1

Means and standard deviations of logarithmized IL-6 and IL-10 levels for all time points during the TSST for PTSD, MDD patients and healthy controls.

|  | PTSD  *Mean (SD)* | MDD  *Mean (SD)* | HC  *Mean (SD)* |
| --- | --- | --- | --- |
|  | IL-6 | | |
| -15 min | 0.76 (0.31) | 0.54 (0.33) | 0.65 (0.24) |
| -1 min | 0.76 (0.27) | 0.47 (0.39) | 0.61 (0.26) |
| +1 min | 0.85 (0.34) | 0.57 (0.28) | 0.64 (0.24) |
| +10 min | 0.82 (0.42) | 0.64 (0.39) | 0.74 (0.27) |
| +20 min | 0.94 (0.41) | 0.68 (0.37) | 0.82 (0.20) |
| +30 min | 0.99 (0.43) | 0.90 (0.46) | 0.82 (0.37) |
| +45 min | 1.02 (0.44) | 0.92 (0.43) | 0.91 (0.34) |
| +60 min | 0.98 (0.41) | 1.09 (0.49) | 1.07 (0.24) |
|  | IL-10 | | |
| -15 min | 0.86 (0.55) | 0.47 (0.21) | 0.36 (0.18) |
| -1 min | 0.76 (0.50) | 0.36 (0.06) | 0.36 (0.18) |
| +1 min | 1.05 (0.52) | 0.42 (0.27) | 0.48 (0.24) |
| +10 min | 0.75 (0.45) | 0.37 (0.15) | 0.41 (0.23) |
| +20 min | 0.79 (0.48) | 0.35 (0.07) | 0.39 (0.23) |
| +30 min | 0.97 (0.46) | 0.34 (0.01) | 0.47 (0.27) |
| +45 min | 1.04 (0.44) | 0.35 (0.15) | 0.39 (0.24) |
| +60 min | 1.08 (0.44) | 0.36 (0.14) | 0.45 (0.19) |

*Notes*. PTSD = Post traumatic stress disorder, MDD = major depression disorder, HC = healthy control, SD = standard deviations, min = minutes.
